# Bilateral Chylothorax due to Lymphatic Reflux Into the Visceral Pleura From Thoracic Duct Obstruction

**DOI:** 10.1002/rcr2.70394

**Published:** 2025-11-03

**Authors:** Minako Shimaya, Yoshiaki Tanaka, Hitoshi Takeuchi, Kozo Yoshimori, Shoji Kudoh

**Affiliations:** ^1^ Department of Internal Medicine Respiratory Disease Center, Japan Anti‐Tuberculosis Association, Fukujuji Hospital Tokyo Japan; ^2^ Department of Radiology Japan Anti‐Tuberculosis Association, Fukujuji Hospital Tokyo Japan

**Keywords:** bilateral chylothorax, lymphangiography, lymphatic reflux, thoracic duct obstruction

## Abstract

A chylothorax associated with lymphatic reflux is extremely rare. A 77‐year‐old woman presented with exertional dyspnea. Chest radiography revealed bilateral pleural effusion, and thoracentesis confirmed chyle. Idiopathic chylothorax was diagnosed as no secondary cause was identified. Lymphangiography revealed thoracic duct obstruction cranial to the hilum. A follow‐up computed tomography scan showed marked accumulation of contrast material in both the lung parenchyma and hilar regions, a pattern suggestive of lymphatic reflux into the lungs. After left‐sided thoracentesis, contralateral pleural effusion decreased, followed by reaccumulation on the left side. These findings indicate an altered chyle distribution after thoracentesis and highlight the role of lymphatic reflux in chylothorax pathogenesis. Recognition of this mechanism may assist diagnosis and guide treatment strategies when no secondary cause can be identified.

## Graphical abstract

We report a rare case of bilateral idiopathic chylothorax that may have resulted from retrograde lymphatic flow into the lungs secondary to thoracic duct obstruction. Lymphangiography and follow‐up CT demonstrated contrast accumulation in the perihilar lymphatic channels and lung parenchyma, supporting the hypothesis of retrograde lymphatic flow rather than direct ductal leakage.

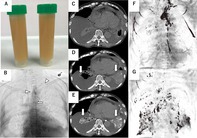

## 1 Introduction

Chylothorax is a rare condition characterised by the accumulation of chylous fluid in the pleural cavity due to disruption of the lymphatic system. Although traumatic and malignant causes are well documented, idiopathic cases present a diagnostic challenge [1]. Lymphangiography is a valuable tool for identifying lymphatic abnormalities; however, reports of reversed lymphatic flow are rare. Here, we report a case of bilateral chylothorax in which thoracic duct obstruction cranial to the pulmonary hilum likely led to abnormal retrograde lymphatic flow.

## 2 Case Report

**FIGURE 1:**
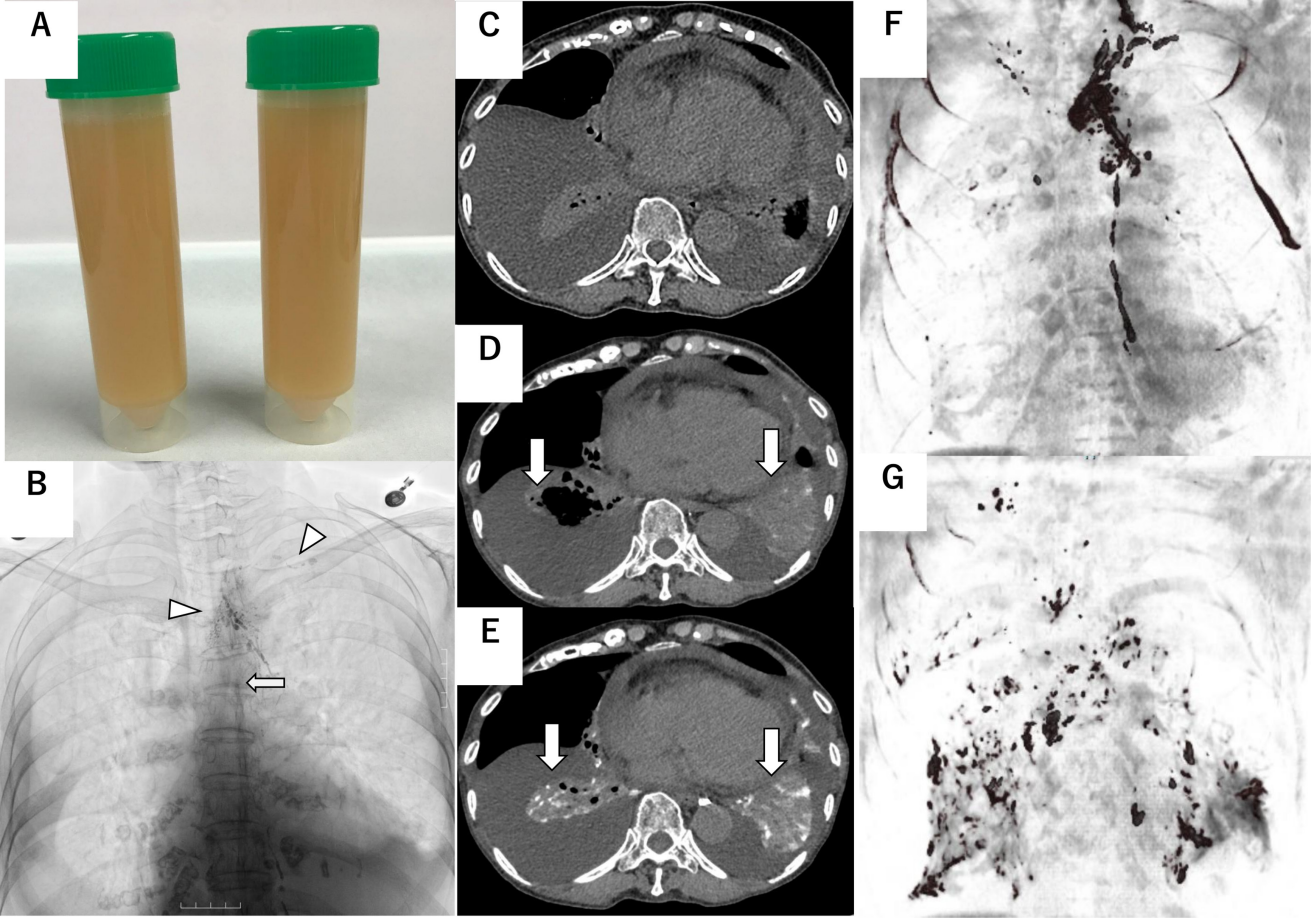
Lymphangiographic and computed tomography (CT) findings with image isolating the contrast‐enhanced structures. (A) Cloudy yellow pleural fluid obtained via thoracentesis. (B) Lymphangiography revealed a thoracic duct obstructed at the level of the carina, cranial to the lung hilum (arrow), without evidence of direct thoracic duct leakage. The contrast medium appears to reflux into lymphatic vessels surrounding the aorta and left subclavian artery (arrowheads). (C) Pre‐lymphangiography CT showing no abnormal high‐attenuation foci. (D) Immediate post‐contrast CT (without intravenous contrast) showing deposition of contrast in the lungs (arrows). (E) Follow‐up CT performed the next day (without intravenous contrast) showing more extensive and intensified bilateral contrast accumulation in the lung parenchyma, particularly near the visceral pleura, compared with the immediate post‐contrast CT (arrows). (F) Contrast‐enhanced structures extracted from the CT shown in (D), highlighting the thoracic duct and mediastinal lymphatic vessels. (G) Contrast‐enhanced structures extracted from the CT shown in (E), demonstrating enhancement of peripheral lymphatic vessels in the perihilar and subpleural regions, whereas the thoracic duct was not visible.

A 77‐year‐old woman presented with a 2‐month history of exertional dyspnea. Her medical history included hypertension and essential thrombocythemia. On examination, she was stable with good oxygenation and no lymphadenopathy. Chest radiography and computed tomography (CT) revealed bilateral pleural effusions without parenchymal abnormalities or lymphadenopathy. Laboratory data were within normal limits except for thrombocytosis. Serum triglyceride and cholesterol levels were 75.1 and 188.2 mg/dL, respectively. Left‐sided thoracentesis yielded milky fluid (Figure 1A) with a triglyceride level of 729.9 mg/dL and a total cholesterol of 77.4 mg/dL, confirming chylothorax. Similar findings were observed on the right side. Lymphangiography was performed via the right inguinal lymph nodes, and approximately 10 mL of lipiodol was injected under fluoroscopic guidance. It demonstrated thoracic duct occlusion at the level of the tracheal bifurcation, anatomically corresponding to the sixth thoracic vertebra, and situated cranial to the pulmonary hilum. Collateral lymphatic flow was visualised around the mediastinal aorta and the left subclavian artery draining into the left venous angle. No contrast leakage from the thoracic duct was observed (Figure 1B). Pre‐lymphangiographic CT (Figure 1C) showed no high‐attenuation foci. However, CT performed 20 min after lymphangiography (Figure 1D) demonstrated scattered high‐attenuation areas in both lungs, which were more pronounced and widespread on follow‐up CT 12 h later (Figure 1E), especially subpleural regions. On contrast‐enhanced reconstruction, the thoracic duct and surrounding lymphatic vessels were visible on an earlier scan (Figure 1F). In contrast, a later image (Figure 1G) showed enhancement in more peripheral lymphatics adjacent to the lungs, along with pulmonary contrast accumulation, whereas the thoracic duct was not visible. No abnormal accumulation was observed in other organs.

**FIGURE 2:**
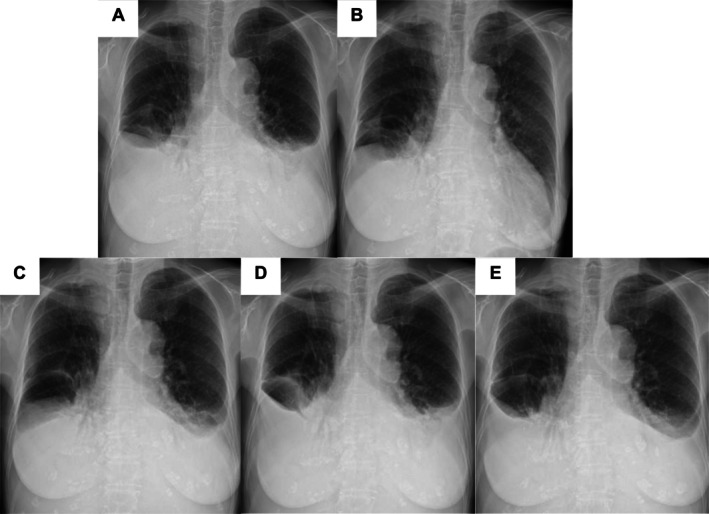
Serial chest radiographs showing the progression of bilateral pleural effusions at five time points. (A) Baseline chest radiograph showing bilateral pleural effusions before intervention. (B) Radiograph after left‐sided pleural drainage showing decreased effusion on the left, whereas the unpunctured right effusion shows a slight decrease. (C) Increased pleural effusion exclusively on the left side without further intervention. (D) Following equilibration of effusion volumes between both sides, a subsequent bilateral increase was observed. These sequential changes shown in (A–D) may reflect redistribution of pleural fluid influenced by intrathoracic pressure dynamics and are consistent with pleural fluid accumulation secondary to retrograde lymphatic flow. (E) Radiograph obtained after initiation of a fat‐restricted diet showing no further increase in effusion volumes, indicating stabilisation.

Before thoracentesis, the pleural effusion volumes were symmetrical (Figure 2A). After drainage of the left‐sided effusion, the size of the unpunctured right effusion decreased slightly (Figure 2B), followed by reaccumulation on the left side only (Figure 2C), and subsequently on both sides (Figure 2D). A similar pattern was observed after right‐sided drainage. These findings suggest that removal of the left pleural effusion redirected chyle flow towards the left side, thereby reducing its inflow into the right pleural cavity. Consequently, chyle absorption through the right parietal pleura likely exceeded the amount entering the cavity, leading to the observed changes.

Based on these findings, reversed lymphatic flow into the lungs was diagnosed as the cause of the chylothorax. This patient had no history of trauma or surgery. Non‐traumatic causes, including cancer, lymphoma, chronic liver disease, medications, lymphatic disorders, chylous ascites, sarcoidosis and infections, were excluded. Given the absence of an identifiable underlying cause, bilateral idiopathic chylothorax was diagnosed. Treatment with therapeutic thoracentesis and a low‐fat diet supplemented with medium‐chain triglycerides was initiated. The pleural effusion did not progress (Figure 2E), and the patient's dyspnea improved.

## 3 Discussion

This case was diagnosed as bilateral idiopathic chylothorax and illustrates a rare mechanism of chylothorax potentially caused by lymphatic reflux. Follow‐up CT performed the day after lymphangiography revealed prominent hyperattenuating foci in the subpleural regions of the lungs, which were more pronounced than those immediately after contrast administration. Evaluation of contrast‐enhanced structures demonstrated accumulation around the thoracic duct immediately following lymphangiography; however, by the following day, the contrast material was predominantly localised to the perihilar lymphatics and lung parenchyma. These findings differ from previously reported cases with thoracic duct injury and overt lymphatic leakage [2]. In this case, although no evidence of thoracic duct injury was observed, the chyle was presumed to have leaked into the pleural cavity through either the visceral or parietal pleura. Several findings support the interpretation that leakage occurred from the visceral pleura. First, no apparent contrast accumulated along the parietal pleura. Second, CT performed approximately 12 h after lymphangiography showed resolution of contrast accumulation along the thoracic duct, with redistribution towards the perihilar region. Third, CT performed 12 h after lymphangiography demonstrated substantial accumulation of contrast material within the lungs. Although this may in part reflect systemic venous return via a collateral pathway caudal to the thoracic duct obstruction of the superior vena cava, this pathway typically results in contrast accumulation in the parietal pleura. However, no enhancement was observed in the parietal pleura, suggesting that the chyle most likely leaked from the visceral pleura. These findings indicate that lymphatic reflux into the lungs plays a central role in chylothorax.

Overall, the chylothorax appears to have resulted from abnormal retrograde lymphatic drainage into the lungs, leading to leakage of chyle from the visceral pleura.

The changes in pleural fluid observed after left‐sided thoracentesis are consistent with a mechanism in which chyle migrates from the mediastinum to the side with lower intrathoracic pressure, supporting the hypothesis that effusion results from retrograde lymphatic flow. Recognition of this mechanism is crucial in cases where conventional causes of chylothorax are not evident and may help guide decisions between conservative and interventional management strategies. The patient responded well to conservative therapy, including dietary fat restriction and intermittent drainage, without progression of pleural effusion for months.

## Author Contributions

M.S. contributed to the conception and drafting of the manuscript. Y.T., H.T., M.S., K.Y. and S.K. contributed to the literature review, critical revision and final approval of the manuscript. All authors approved the final version.

## Consent

The authors declare that written informed consent was obtained for the publication of this manuscript and the accompanying images and attest that the consent form complies with the journal's requirements as outlined in the author guidelines.

## Conflicts of Interest

The authors declare no conflicts of interest.

## Data Availability

The data that support the findings of this study are available from the corresponding author upon reasonable request.
